# A Window into the Workings of the Segmentation Clock

**DOI:** 10.1371/journal.pbio.1001366

**Published:** 2012-07-24

**Authors:** Janelle Weaver

**Affiliations:** Freelance Science Writer, Glenwood Springs, Colorado, United States of America

[Fig pbio-1001366-g001]Rhythms underlie a range of biological phenomena, from circadian clocks to cellular responses to DNA damage. The formation of body parts is no exception. During development, the cyclical expression of genes is crucial for regulating the sequential formation of body segments called somites in vertebrates, including zebrafish. Members of the *hes*/*her* family of genes are expressed in a rhythmic fashion during this process, and the oscillations of this genetic network, known as the segmentation clock, guide segmentation and control the speed at which somites form, as well as the number of somites. Models for the origin of oscillations in the zebrafish segmentation clock propose a negative feedback loop involving the *her1* and *her7* genes. But biochemical evidence supporting this model has been lacking, and exactly how these genes interact to control segmentation and its timing in the zebrafish embryo has been a long-standing mystery.

**Figure 1 pbio-1001366-g001:**
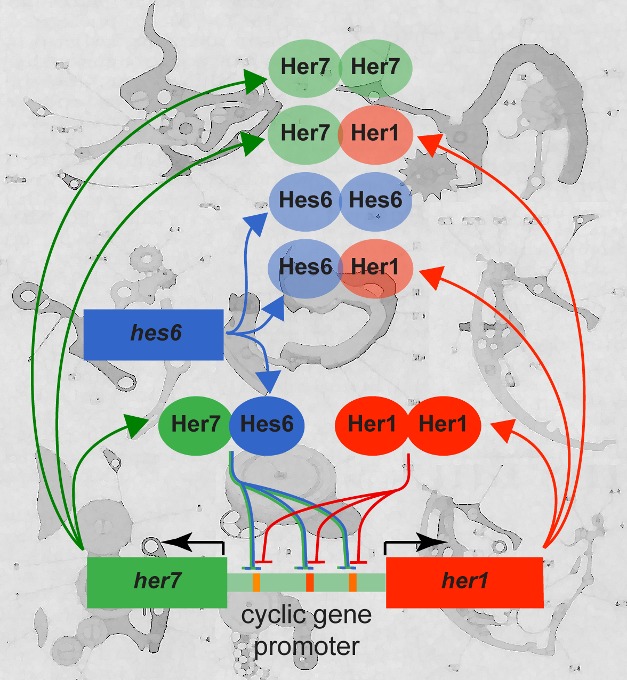
How timing directs anatomy: the pacemaking circuits at the core of the zebrafish embryo's segmentation clock contain two negative feedback loops, consisting of Her1:Her1 and Her7:Hes6 protein complexes.

In this issue of *PLoS Biology*, a team led by Andrew Oates of the Max Planck Institute of Molecular Cell Biology and Genetics has shed new light on the genetic network that regulates segmentation in zebrafish. The researchers found that two negative feedback loops, consisting of Her1:Her1 and Her7:Hes6 protein complexes, make up the core pace-making circuit of the segmentation clock. The existence of two co-existing loops may make this important developmental process robust in the face of genetic and environmental disturbances.

In a series of biochemical experiments, Oates and his collaborators found that Her1, Her7, and Hes6 pair up to form all sorts of protein complexes, but only Her1:Her1 and Her7:Hes6 complexes bind to DNA to regulate the expression of cyclic genes—those that are expressed in an oscillating pattern. These two types of complexes bind to the same DNA sequences to regulate the cyclic genes *her1*, *her7*, and *dlc*.

Next, the researchers tested the function of the Her1:Her1 and Her7:Hes6 feedback loops in zebrafish. In embryos that were genetically modified to lack either *hes6* or *her1*, cyclic genes were expressed in an oscillatory fashion and segmentation was mostly normal because the intact loop was able to compensate for the disrupted one. By contrast, when they simultaneously impaired both loops by disrupting both *her1* and *hes6*, or *her1* and *her7*, gene expression did not oscillate and the embryos did not segment normally. These results suggest that the two redundant, parallel feedback loops form the core circuit of the segmentation clock in zebrafish.

But the two-loop model is more complicated than expected. Surprisingly, zebrafish with *her7* mutations did not segment normally, and these defects disappeared for the most part when *her7* mutations existed alongside *hes6* mutations. Mathematical models suggested that in the absence of Her7, more Hes6 is free to bind to Her1, thereby reducing the number of Her1:Her1 complexes. This process interferes with the Her1:Her1 feedback loop, dampens oscillatory gene expression, and causes segmentation defects. These results suggest that Hes6 plays a dominant role in guiding segmentation, depending on the availability of the protein's binding partners. In addition, mathematical models indicated that Hes6 changes the stability of oscillating proteins, and this process influences the speed of segmentation.

The delicate balance of Hes/Her proteins required for the segmentation clock to function properly is similar to that seen in the genetic network controlling the mouse circadian clock. Moreover, the Hes/Her family of cyclic genes is involved in segmentation in mice and chickens. Thus, the new principle for the segmentation clock may underlie multiple biological clocks and genetic regulatory networks in a range of species.

**Schröter C, Ares S, Morelli LG, Isakova A, Hens K, et al. (2012) Topology and Dynamics of the Zebrafish Segmentation Clock Core Circuit. doi:10.1371/journal.pbio.1001364**

